# Genome-Wide Identification of the Alfin-like Gene Family in Cotton (*Gossypium hirsutum*) and the *GhAL19* Gene Negatively Regulated Drought and Salt Tolerance

**DOI:** 10.3390/plants13131831

**Published:** 2024-07-03

**Authors:** Jie Liu, Zhicheng Wang, Bin Chen, Guoning Wang, Huifeng Ke, Jin Zhang, Mengjia Jiao, Yan Wang, Meixia Xie, Qishen Gu, Zhengwen Sun, Liqiang Wu, Xingfen Wang, Zhiying Ma, Yan Zhang

**Affiliations:** State Key Laboratory of North China Crop Improvement and Regulation, North China Key Laboratory for Germplasm Resources of Education Ministry, Hebei Agricultural University, Baoding 071001, China

**Keywords:** genome-wide characterization, Alfin-like TF, *Gossypium hirsutum*, abiotic stresses, ABA-mediated pathway

## Abstract

Alfin-like (AL) is a small plant-specific gene family characterized by a PHD-finger-like structural domain at the C-terminus and a DUF3594 structural domain at the N-terminus, and these genes play prominent roles in plant development and abiotic stress response. In this study, we conducted genome-wide identification and analyzed the AL protein family in *Gossypium hirsutum* cv. NDM8 to assess their response to various abiotic stresses for the first time. A total of 26 AL genes were identified in NDM8 and classified into four groups based on a phylogenetic tree. Moreover, cis-acting element analysis revealed that multiple phytohormone response and abiotic stress response elements were highly prevalent in AL gene promoters. Further, we discovered that the GhAL19 gene could negatively regulate drought and salt stresses via physiological and biochemical changes, gene expression, and the VIGS assay. The study found there was a significant increase in POD and SOD activity, as well as a significant change in MDA in VIGS-NaCl and VIGS-PEG plants. Transcriptome analysis demonstrated that the expression levels of the ABA biosynthesis gene (*GhNCED1*), signaling genes (*GhABI1*, *GhABI2*, and *GhABI5*), responsive genes (*GhCOR47*, *GhRD22*, and *GhERF*s), and the stress-related marker gene *GhLEA14* were regulated in VIGS lines under drought and NaCl treatment. In summary, *GhAL19* as an AL TF may negatively regulate tolerance to drought and salt by regulating the antioxidant capacity and ABA-mediated pathway.

## 1. Introduction

Drought and salt are unfortunate conditions for agriculture; in fact, drought alone causes more annual crop yield loss than all pathogens combined [1]. On the one hand, climate change is leading us towards a hotter and more parched world [1]. On the other hand, climate warming causes sea levels to rise, which also leads to large areas of land salinization; therefore, there is an urgent need to develop high-yielding plants that can tolerate salt and drought [2]. Cotton is the most important natural fiber crop globally and is particularly susceptible to the negative impacts of drought and salt. These stresses can severely affect the growth, development, and yield of cotton, leading to significant losses [3,4]. Transcription factors can improve the adaptability of plants to adverse environments like cold, salt, drought, and pathogens [5].

The AL protein family has been found to act as a transcription factor family in alfalfa (*Medicago sativa*) with a 7S storage protein [6]. Members of this family have two conserved amino acid sequences of approximately 130 and 50 bp at N-termini and C-termini, known as the DUF3594 domain and PHD-finger motif [6]. Most AL proteins can bind to the conserved cis-element GNGGTG/GTGGNG through the N domain, and the PHD-finger motif can bind to the core consensus cis-acting element (C/A) CAC in the promoter of the target gene [7]. The study finds that proteins containing the PHD domain, like alfin1 orthologs, have been shown to bind to the promoters of H3K4me2 and H3K4me3, suggesting their involvement in the process of chromatin regulation in both plants and animals [8]. Comprehensive molecular evolutionary analyses have been conducted in two Arabidopsis species (*Arabidopsis lyrata* and *Arabidopsis thaliana*) and a close salt-tolerant relative, *Thellungiella halophila*. The results indicate that positive selection operates on four branches of AL genes, and significant differences in biased codon usage in the AL family are between *T. halophila* and *A. lyrate/A. thaliana*. And salt-stress-induced *AtAL7* of *A. thaliana* can play a negative role in salt tolerance, suggesting that adaptive evolution occurred in the members of the AL gene family [9]. More and more studies have discovered that the AL gene can be involved in abiotic resistance, such as over-expressing *MsAL1* genes that can promote root growth under normal and high salt conditions, as well as enhance the salt tolerance of alfalfa (*Medicago sativa L.*) [7,10,11,12]; the *GmPHD2* gene with the PHD motif showed higher salt tolerance in the transgenic Arabidopsis with *GmPHD2* [13]; and *AtAL5* gene enhances cold, salt, and drought tolerance by regulating the expression of related downstream genes [14]. Moreover, *AhAL1* of *Atriplex hortensis* transferred into Arabidopsis enhances the drought resistance and salt tolerance of Arabidopsis [15]. Additionally, the majority of AL genes in poplar are strongly expressed in response to abiotic conditions such as low temperature, high temperature, salt (100 mM NaCl solution), and drought (water holding), of which *PtAL4* and *PtAL6* are sensitive to all evaluated abiotic stressors, while *PtAL5* and *PtAL7* can be used in the populous breeding program to generate stress-resistant cultivars [16]. Analysis of the *SlAL3* gene in tomatoes also reveals that its expression can be induced by drought and salt stress [17]. Furthermore, the AL gene family in *Brassica rapa* and *Brassica oleracea* is also found to respond to cold, drought, and salt stress [18,19]. These all indicate that the AL gene family is involved in abiotic tolerance and displays divergent functions during evolution. Recent studies on abscisic acid (ABA) signaling have revealed that the detailed molecular mechanisms by the various proteins (such as transporters, receptors, signaling regulators, and transcription factors) function to activate ABA responses in plants during drought stress [20,21,22,23]. In *A*. *hortensis*, ABA-mediated stomatal closure, inhibition of seed germination, and primary root elongation are all enhanced in *AhAL1*-transgenic plants [15]. However, the role of the AL gene family in cotton abiotic stress responses and whether they are regulated by ABA signaling remain unknown.

In this study, an AL transcription factor, *GhAL19*, has been identified from NDM8, which is responsive to drought and salt stress. In detail, the *GhAL19* gene can be induced by drought and salt stresses, and VIGS-*GhAL19* cotton can enhance tolerance to those stresses and affect gene expression related to the ABA signal pathway.

## 2. Results

### 2.1. Genome-Wide Identification of Alfin-like Genes in Cotton

To identify all AL genes in NDM8, we conducted a BLASTP search against the tetraploid cotton protein databases using the AL sequences of Arabidopsis (7 AL genes in Arabidopsis) as queries. Then the potential AL proteins in cotton were analyzed using the SMART databases to confirm the AL protein domain (PF12165). Eventually, 26 AL members were identified in NDM8. These AL genes were named from *GhAL1* to *GhAL26* based on the order of their chromosomal locations (Table 1). Further, 24 of the 26 GhAL genes encoded proteins ranging between 225 and 291 amino acids (aa), except for 2 genes with lengths of less than 213 (i.e., *GhAL24* encodes 184 aa, and *GhAL5* encodes 176 aa). The predicted Mw (molecular weight) of GhAL proteins ranged from 20.02 to 32.82 kDa, and the theoretical pI varied between 4.374 and 6.474. Physicochemical analysis revealed that all GhAL proteins in NDM8 were acidic and had low isoelectric points (pI < 7). According to the protein subcellular localization of GhAL in cotton predicted by Cell-PLoc 2.0, almost all AL proteins were predicted to be located in the nucleus, with *GhAL5* in the cytoplasm and extracellular and *GhAL1* in the nucleus and extracellular (Table 1). The above results suggested that GhAL genes have a functional basis as a transcription factor except *GhAL5*.

### 2.2. Phylogenetic Analysis of the Alfin-like Gene Family

**Figure 1:**
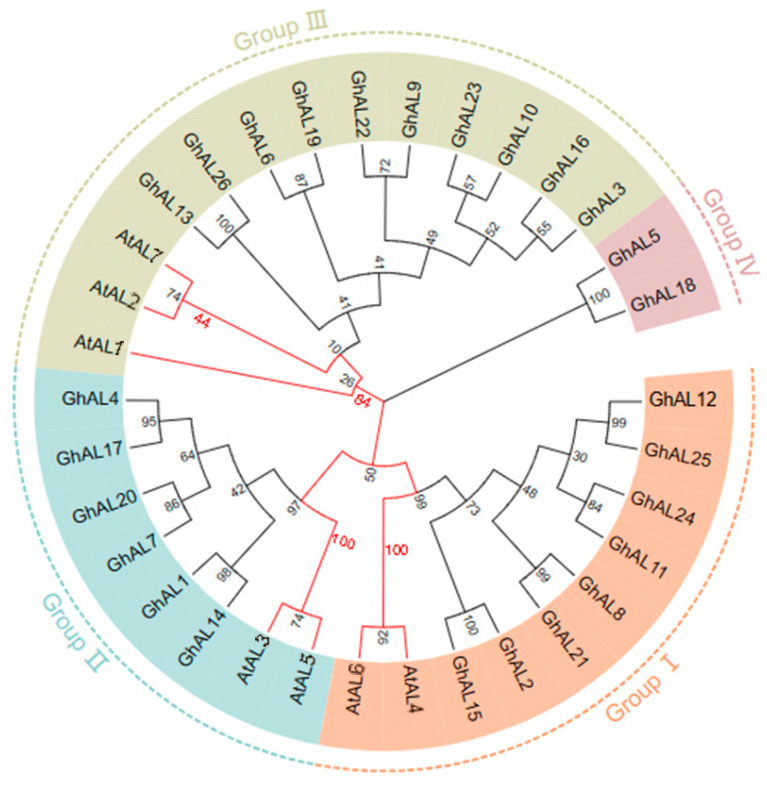
Phylogenetic analysis of the Alfin-like family proteins in NDM8 and *Arabidopsis thaliana*. The phylogenetic tree was constructed by the neighbor-joining method using MEGA. Bootstrap values were 1000 repeats for the main branches. The subgroup was divided according to the red values on the node at the root of the evolutionary tree of *A. thaliana* (the value ≥ 80: all genes under this node belong to one subgroup; the value < 80: all genes under this node can be further divided into two or more subgroups). Alfin-like family genes in NDM8 were divided into four subfamilies according to *A. thaliana.* Orange-Group I; Blue-Group II; Green-Group III; Pink-Group VI. Accession numbers are as following: *AtAL1* (*NP_196180.1*), *AtAL2* (*NP_187729.1*), *AtAL3* (*NP_189865.1*), *AtAL4* (*NP_197993.1*), *AtAL5* (*NP_197551.2*), *AtAL6* (*NP_178351.1*), *AtAL7* (*NP_172* NDM8*903.1*).

To assess the phylogenetic relationships of AL genes among NDM8 and *A. thaliana*, we constructed a phylogenetic tree using the NJ method (Figure 1). The phylogenetic tree was also reconstructed using the maximum likelihood (ML) method, which was almost identical with only minor differences at some branches compared to the NJ method (Figure S1). This suggested that the two methods were largely consistent with each other and the results of the evolutionary tree were accurate. As shown in Figure 1, GhAL genes could be divided into four groups: Group I (including 8 genes), Group II (6), Group III (10), and Group IV (2). The two AL genes in Group IV did not have the orthologous gene in Arabidopsis. The observed evolutionary relationships indicate that most of the AL proteins in cotton were closely related to AtAL proteins (Figure 1).

### 2.3. Gene Structure and Domain Analysis

**Figure 2:**
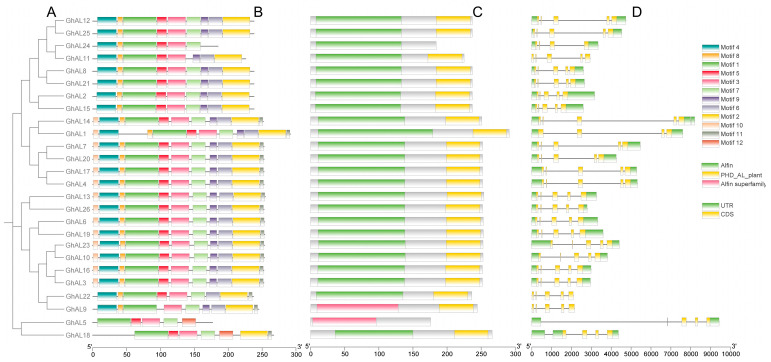
Conserved motifs, gene structures, and conserved domains of Alfin-like family members. (**A**) The phylogenetic tree on the left was constructed with the program MEGA 7.0 using full-length amino acid sequences by the Neighbor-Joining (NJ) method with 1000 bootstrap repeats. (**B**) The prediction of conserved, novel, and untapped motifs in AL protein sequences. The motifs were predicted using the MEME online server. The different conserved motifs were marked by different colors. The size of proteins can be estimated using the scale at the bottom. (**C**) Conserved domains of AL proteins: the green/yellow rectangle represents the *AL* conserved domain containing the Alfin domain and PHD domain. The size of proteins can be estimated using the scale at the bottom. (**D**) Exon/intron organization of *AL* genes. Exons and introns are indicated by yellow boxes and black lines, and the untranslated regions (UTRs) are indicated by green boxes. The sizes of exons and introns can be estimated using the scale at the bottom.

To gain insight into the gene structure of the AL family in cotton, we aligned the full-length protein sequence of 26 AL genes to create a phylogenetic tree (Figure 2A) with conserved motifs, exon-intron, and domain structures. We identified 12 different motifs in the AL protein sequences by using the MEME online service. These motifs were distributed throughout the sequences, with each protein containing five or eleven motifs (Figure 2B). Further analysis revealed that all 26 AL proteins had high similarity; for example, motif 1, motif 3, and motif 5 were present in each AL protein and likely constituted the AL domain. However, each AL subgroup contained multiple specific domains, such as motif 10, which was absent in Group I. Notably, *GhAL5* and *GhAL18*, which did not have an orthologous gene in *A. thaliana*, had fewer motifs compared to others, lacking motif 4, motif 6, motif 8, motif 9, and motif 10, but they possessed a new motif 12.

The domain structure of AL proteins was analyzed using NCBI CDD to predict conserved domains of the AL family. The result showed that the members of AL had a conserved Alfin domain (about 120 amino acids) and a conserved PHD-finger domain (about 60 amino acids). These domains were connected by a variable region of approximately 80 amino acids at the N and C terminals (Figure 2C). However, *GhAL5* and *GhAL24* lacking the PHD domain might result in a decrease in their binding ability to DNA, RNA, or proteins, or the loss of certain functionalities in NDM8 (Figure S2). Meanwhile, it was also observed that the two genes did not have motif 2 (Figure 2B). In conclusion, members belonging to the same subfamilies of the phylogenetic tree had a similar gene structure and conserved motif, consistent with the results of the phylogenetic analysis.

We also conducted exon/intron gene structure analysis by comparing genomic sequences. GhAL genes all had four introns and five exons, except *GhAL5* and *GhAL18*. However, the length of the introns of the AL genes varied significantly (Figure 2D). Furthermore, *GhAL11*, *GhAL9*, and *GhAL22* lacked 3′ and 5′ UTR sequences. Intron/exon structure analysis of BrAL and AtAL TFs revealed that all ALs had four introns and five exons, which was consistent with the number of exons and introns in cotton AL TFs [14,18]. This conserved exon/intron structure between species supported their close evolutionary relationship and the classification of the groups.

### 2.4. Genomic Localization and Collinearity Analysis

To investigate the distribution and gene duplication of the AL family genes in cotton, we conducted chromosomal distribution and collinearity analyses. We mapped the AL genes onto chromosomes in NDM8 using available genomic information (Figure S3). A total of 26 AL genes were found on 14 chromosomes, of which four AL genes were found on chromosomes D06 and A06 respectively, two AL genes were found on chromosomes A05, A10, A13, D05, D10, and D13 respectively, and one AL gene were found on chromosomes A03, A04, A07, D02, D04, and D07, respectively.

**Figure 3:**
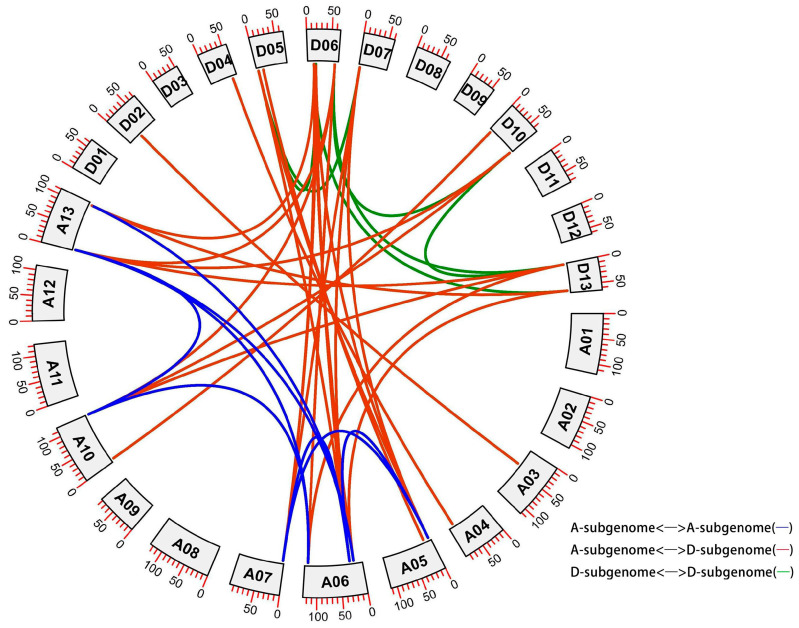
Genomic localization and collinearity analysis of GhAL genes. A01 to A13 represent A-subgenome chromosomes, while D01 to D13 represent D-subgenome chromosomes. Homologous gene pairs between the A- and A-subgenomes were 7 pairs and represented with blue lines; homologous gene pairs between the A- and D-subgenomes were 24 pairs and represented with red lines; and homologous gene pairs between the D- and D-subgenomes were 5 pairs and represented with green lines.

To examine the locus relationships between the A- and D-subgenomes of the cotton, we performed a collinearity analysis (Figure 3) and analyzed gene duplication variety. The details of the duplicated gene pairs can be found in Table S1. We identified a total of 36 paralogous gene pairs in NDM8 with a Ka/Ks ratio < 1 (Figure 3 and Table S1). Of which, homologous gene pairs between the A- and A-subgenome were 7 pairs, between the A- and D-subgenome were 24 pairs, and between the D- and D-subgenome were 5 pairs, suggesting that gene replication occurs mostly among the A- to D-subgenome. Specifically, all the duplicated AL gene pairs had a Ka/Ks ratio < 1, indicating that they had experienced purifying selection pressure with limited functional divergence (Table S1). This reflected that the functions of the duplicated GhAL genes in NDM8 had not significantly diverged during subsequent evolution, and purifying selection had played a crucial role in maintaining the function of the GhAL genes.

### 2.5. Expression Patterns of AL Genes in Upland Cotton

**Figure 4:**
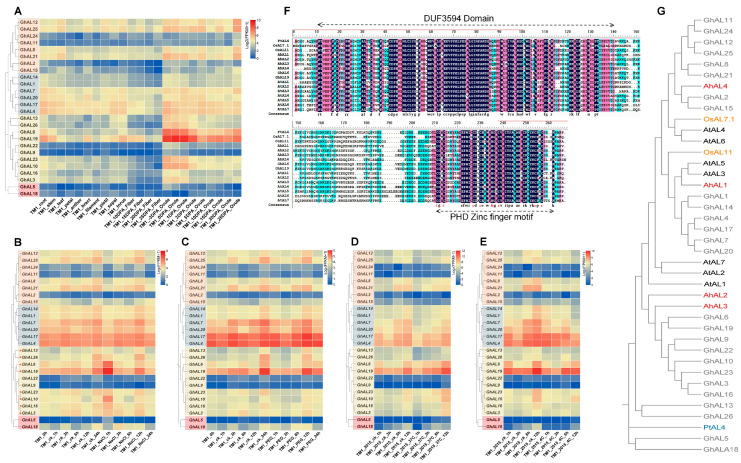
Expression patterns of the *AL* gene in cotton and evolutionary and conserved domains of the *AL* gene in cotton and other plant species. (**A**) Expression patterns of AL genes in different tissues of TM-1. (**B**–**E**) Expression patterns of AL genes in different stress treatments. The raw data for RNA-Seq of TM-1 was downloaded and used to analyze the expression patterns of *AL* genes. The color bar represents the expression values in log_2_(TPM + 1). Alfin-like family genes in cotton were divided into four subfamilies according to *A. thaliana.* Orange-Group I; Blue-Group II; Yellow-Group III; Pink-Group VI. The red star in B refers to the gene in Group III. (**F**) Alignment of the conserved Alfin domain and the PHD domain of the *GhAL19* and AL genes in Poplar, Rice, *A. thaliana*, and *A. hortensis*. The C_4_HC_3_ zinc-finger motifs are indicated by red words. The high consensus color was black, the low consensus color was red and blue, and the neutral color was white. (**G**) The phylogenetic tree of AL genes in TM-1 and AL genes in Poplar, Rice, *A. thaliana*, and *Atriplex hortensis*. Accession numbers are as follows: *AhAL1* (*KU933956*), *AhAL2* (*KY322832*), *AhAL3* (*KY322833*), *AhAL4* (*KY322834*); *PtAL4* (*Potri.006G145300*); *OsAL11* (*LOC_Os11g14010*), *OsAL7.1* (*LOC_Os07g12910*); *AtAL1* (*NP_196180.1*), *AtAL2* (*NP_187729.1*), *AtAL3* (*NP_189865.1*), *AtAL4* (*NP_197993.1*), *AtAL5* (*NP_197551.2*), *AtAL6* (*NP_178351.1*), *AtAL7* (*NP_172* NDM8*903.1*).

In order to understand the temporal and spatial expression patterns of upland cotton AL genes, we analyzed their expression profiles in different tissues using publicly available expression data from cottonMD. We extracted RNA-seq data of AL genes from various tissues, developmental stages, and different times under stress in *G. hirsutum* cv. TM-1. All values in Figure 4A were subjected to log_2_(FPKM + 1). The results illustrated that AL genes in TM-1 exhibited diverse expression patterns in different tissues. Ten AL genes (*GhAL4*, *GhAL6*, *GhAL7*, *GhAL8*, *GhAL12*, *GhAL17*, *GhAL19*, *GhAL20*, *GhAL21*, and *GhAL25*) in TM-1 could be detected in all the tested tissues. Notably, *GhAL6* and *GhAL19* showed high accumulation levels during ovule development (−3 to 25 DPA). As we know, long lint fibers were produced before or on the day of anthesis (i.e., 0 DPA), while ovule epidermal cells started at or after 3 DPA and produced shorter fibers. The elongation of fuzz fibers began at 5–10 DPA in *G. hirsutum* [24,25], indicating that these two genes might play significant roles in ovule and fiber development and are likely involved in regulating fiber formation.

### 2.6. Expression Patterns under Salt, Drought, and Temperature Stresses

Previous studies have reported that the AL genes of *A. thaliana*, *Oryza sativa L.*, *Populus trichocarpa*, and *A. hortensis* were associated with plant stress resistance [9,15,16,26]. For example, overexpression of *OsAL7.1* and *OsAL11* in rice weakens drought tolerance in the adult stage. *PtAL4* gene expression decreases with longer durations of drought, cold, and salt treatments but increases after heat treatment [15,16,27]. However, the function of AL genes in cotton responses to salt or cold stress remains unknown.

To better understand the function of GhAL under abiotic stresses in *G. hirsutum*, we presented the expression pattern of GhAL genes in response to stresses of salt, PEG, 4 °C, and 37 °C using the FPKM values from CottonMD (Transcriptome data from WHU). The expression of six AL genes (*GhAL2*, *GhAL5*, *GhAL9*, *GhAL11*, *GhAL22*, and *GhAL24*) was very low and slightly affected by NaCl (Figure 4B). The expression of the other 20 AL genes was upregulated under NaCl treatment at 12 h. Of which, the 8 genes in Group III (*GhAL3*, *GhAL6*, *GhAL10*, *GhAL13*, *GhAL16*, *GhAL19*, *GhAL23*, and *GhAL26*) were up-regulated 2–7 folds under NaCl treatment for 1 h. The expressions of AL genes under PEG treatment were almost upregulated at 12 h and downregulated at 24 h, especially the genes in Group III (Figure 4C). There was no significant change in gene expression in Group I and Group II when plants were exposed to high temperatures, while the *GhAL6* and *GhAL19* in Group III were upregulated 1.5 folds at 12 h under 37 °C (Figure 4D). However, the expressions of all AL genes were downregulated when exposed to cold stress (Figure 4E).

To investigate the stress resistance of AL genes in cotton, we further analyzed the AL gene family of cotton along with the published abiotic stress-related AL genes of *A. thaliana*, *O. sativa L.*, *P. trichocarpa*, and *A. hortensis* (Figure 4F,G). The results suggested that the AL genes in Group III could be closely related to drought or salt tolerance, especially *GhAL19*, which could be induced by drought, salt, and temperature. Therefore, we designed silencing experiments for this gene to verify its function in the following experiments.

Furthermore, the cis-elements of these AL gene promoters were analyzed with PlantCARE, which could provide critical evidence and understanding of the gene functions [28]. Then the promoter regions up to 2 kb upstream of all 26 AL genes were analyzed (Figure S4). The result showed that 14 putative environmental stimulus-responsive cis-elements were found in AL genes, and these elements were divided into two types, including stress-responsive elements (AS-1, AC-1, ARE, DRE1, LTR, MBS, MYB, MYC, TC-rich repeats, GC-motif, WRE3, W-box, STRE, and WUN-motif), and hormone-responsive elements (ABRE, ERE, GARE, P-box, CGTCA-motif, TGACG-motif, and TGA-element). This suggests that AL genes may play a key role in regulating plant responses to various abiotic stresses and plant growth.

### 2.7. GhAL19 Regulates Salt, Drought, and Hot Stress and Increases Reactive Oxygen Species Metabolic Process

**Figure 5:**
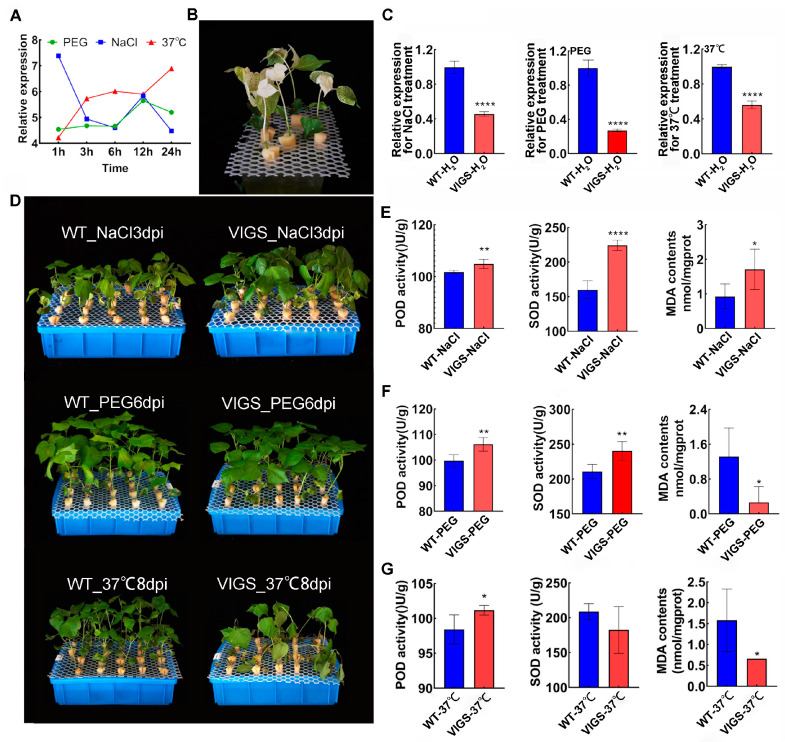
Phenotypic identification and physiological indexes of the *GhAL19* VIGS line under salt, drought, and hot stress. (**A**) The expression levels of *GhAL19* under NaCl/PEG/37 °C treatment at 1 h, 3 h, 6 h, 12 h, and 24 h. (**B**) The plants with the CLA1 gene show an albino phenotype on newly grown leaves and stems. (**C**) The relative expression levels of *GhAL19* in WT-H_2_O and VIGS-H_2_O lines for different treatments. Each qPCR reaction was performed with four technical replicates. (**D**) Phenotypic identification of VIGS-treatment and WT-treatment. And WT/VIGS cotton has been exposed to NaCl for 3 days (NaCl3dpi), WT/VIGS cotton has been exposed to PEG for 6 days (PEG6dpi), and WT/VIGS cotton has been exposed to 37 °C for 8 days (37 °C8dpi). (**E**–**G**) SOD activity, POD activity, and MDA content in the VIGS-treatment and WT-treatment plants (the treatments are NaCl3dpi, PEG6dpi, and 37 °C8dpi, respectively). The symbol ‘*’ indicates the significance of the difference, of which ‘*’ represents *p* < 0.05, ‘**’ represent *p* < 0.01, ‘****’ represent *p* < 0.0001.

Salt, temperature, and water stresses are the main factors that significantly inhibit crop growth and lead to a decline in yield [1]. A new tolerance gene, *GhAL19*, has been mined and is affected by salt, PEG6000, and 37 °C conditions (Figure 5A). Subsequently, the function of *GhAL19* under those conditions was determined by using the VIGS (virus-induced gene silencing) system. When an albino-like appearance was observed on the first true leaf (Figure 5B), the expression levels of *GhAL19* in WT cotton with no treatment (WT-H_2_O) and VIGS-*GhAL19* lines (VIGS-H_2_O) were examined. And the expression levels of *GhAL19* were significantly decreased in each VIGS line compared to the corresponding WT-H_2_O (Figure 5C). Then the cotton plants (WT-H_2_O and VIGS-H_2_O) were exposed to NaCl, PEG6000, and 37 °C stresses, respectively. We found that the silenced *GhAL19* gene enhanced the tolerance to salt and drought treatments compared to WT-NaCl and WT-PEG, respectively, but reduced the tolerance to hot treatment (Figure 5D) compared to WT-37 °C. Interestingly, the VIGS plants exhibited a salt-tolerant phenotype on the third day, a drought-resistant phenotype on the sixth day, and a sensitivity to 37 °C on the eighth day. The results indicated that the regulation and response of *GhAL19* to different abiotic stresses within a certain dosage might have a specific time series.

In addition, we further detected the activities of antioxidant enzymes and the content of malondialdehyde (MDA) in the WT-treatment (NaCl/PEG/37 °C) and VIGS-treatment (NaCl/PEG/37 °C) lines. The results showed a significant increase in peroxidase (POD) activity and a decrease in the content of malondialdehyde (MDA) in VIGS-PEG plants and VIGS-37 °C plants (Figure 5E,G). While there was a significant increase in POD and superoxide dismutase (SOD) activity, as well as an increase in MDA content, in VIGS-NaCl cotton plants (Figure 5F). Taken together, *GhAL19* may positively regulate hot tolerance and negatively regulate salt and drought tolerance in NDM8 by regulating the antioxidant capacity of cotton.

### 2.8. GhAL19 May Act as a Metabolite Hub Regulated Drought and Salt Resistance

**Figure 6:**
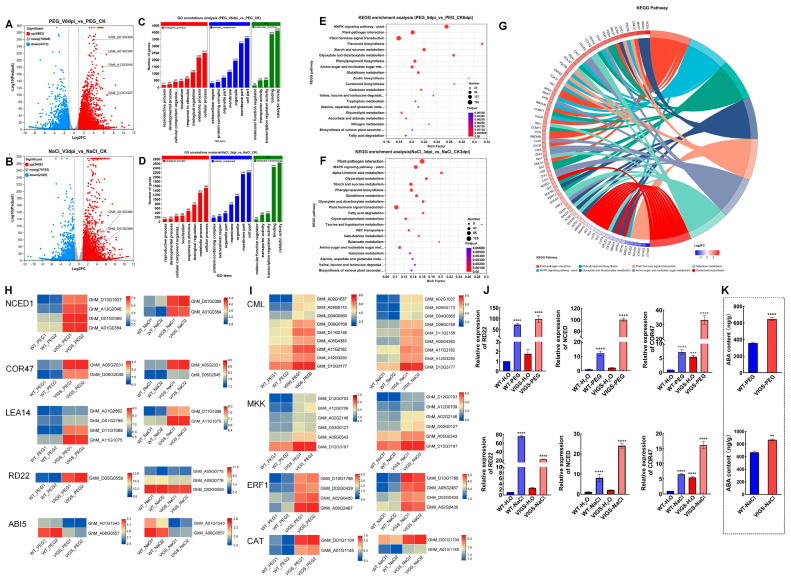
Transcriptome profiles of genes in the VIGS-GhAL19 and WT cotton under drought and salt stress. (**A**,**B**) Volcano map of DEGs in PEG and NaCl treatments (VIGS-treatment vs. WT-treatment), respectively. *GhM_D01G0369*, *GhM_A01G0384*, *GhM_A13G2046*, *and GhM_D13G1937* are NCED1 genes. (**C**,**D**) GO annotation analysis of DEGs in PEG and NaCl treatments, respectively. (**E**,**F**) KEGG enrichment analysis of DEGs in PEG and NaCl treatments, respectively. (**G**) KEGG pathway enrichment diagram of important genes related to resistance and pathways after PEG and NaCl treatments. Of which, gene names are on the left of the circle; KEGG Pathway description information is on the right of the circle; and log_2_FC is the logarithmic value of the gene/transcript at base 2 of the multiple of the difference between two samples. (**H**,**I**) Heatmap of the important genes related to resistance in PEG and NaCl treatments, and the values were normalized by the average log_2_(TPM + 1). (**J**) Expression of *GhNCED1*, *GhRD22*, and *GhCOR47* in WT-H_2_O, WT-treatment, VIGS-H_2_O, and VIGS-treatment lines. WT-H_2_O refers to control cotton without any treatment, and WT-PEG refers to control cotton with PEG treatment after 6 h. WT-NaCl refers to control cotton with NaCl treatment after 3 h. VIGS-H_2_O refers to VIGS-*GhAL19* cotton without any treatment. VIGS-PEG refers to VIGS-*GhAL19* cotton with PEG treatment after 6 h. VIGS-NaCl refers to VIGS-*GhAL19* cotton with NaCl treatment after 3 h. (**K**) ABA content of WT-treated cotton and VIGS-treated cotton, respectively. The symbol ‘*’ indicates the significance of the difference, of which ‘**’ represent *p* < 0.01, ‘***’ represent *p* < 0.001, ‘****’ represent *p* < 0.0001.

To identify the signaling pathways potentially under the transcriptional regulation of *GhAL19* in response to salt and drought, we conducted RNA-seq analyses to examine genome-wide gene expression in WT-treated (NaCl and PEG) and VIGS-treated (NaCl and PEG) cotton. The results showed there were 3606 genes upregulated and 2325 genes downregulated after salt treatment, while 4863 genes were upregulated and 4313 genes were downregulated after drought treatment (Figure 6A,B). In VIGS-*GhAL19* cotton plants, the differentially expressed genes (DEGs) were enriched in various categories, including cell process/metabolic process, cell part/membrane part, and catalytic activity/binding according to GO annotation (Figure 6C,D). Additionally, KEGG enrichment analysis showed that the DEGs were significantly enriched in pathways related to MAPK signal pathway-plant, plant hormone signal transduction, phenylpropanoid biosynthesis, carotenoid biosynthesis, and plant-pathogen interaction (Figure 6E,F).

In more detail, four dominant expression genes involved in carotenoid biosynthesis (*NECD*, 9-cis-epoxy carotenoid dioxygenase, *GhM_A01G0384*, *GhM_D01G0369*, *GhM_D13G1937*, and *GhM_A13G2046*) were significantly upregulated tenfold to a hundredfold in VIGS-PEG cotton compared to WT-PEG (Figure 6A). And, two genes *GhM_D01G0369* and *GhM_A01G0384* were upregulated 2 times in VIGS-NaCl cotton. The ABA signaling genes *ABI1* (*GhM_A06G0795*, *GhM_D06G0795* and *GhM_A13G2575*) and *ABI2* (*GhM_D13G2489*, *GhM_D08G3076*, *GhM_D07G0153* and *GhM_A07G0154*) were significantly up-regulated by 3–6 times in VIGS lines compared to WT-treatments. The ABA-responsive genes *COR47* that response to water deprivation (*GhM_A05G2031* and *GhM_D05G2045*) and *RD22* (*GhM_D05G0559*) and stress-related marker genes *LEA14* (*GhM_A11G1075* and *GhM_D11G1088*) were also upregulated in silence cotton plant under salt and drought stresses (Figure 6G,H). Moreover, the expression of nearly 30 *ERF* genes (ethylene-response factor, Figure S5A) increased under stress conditions. ERFs are known to be key regulators of various stress responses, and they are responsive to the plant hormones abscisic acid (ABA) and ethylene (ET) [29]. ERFs also help activate ABA and ET-dependent and -independent stress-responsive genes [29]. More DEGs, including CML (calcium-binding protein, *GhM_A02G1037*, *GhM_A09G0173*, *GhM_D04G0560*, *GhM_D09G0169*, *GhM_D11G2156*, *GhM_A05G4383*, *GhM_A11G2182*, *GhM_A12G3290*, *GhM_D12G3177*), MPK (mitogen-activated protein kinase7, *GhM_D12G0703*, *GhM_A12G0709*, *GhM_A02G2146*, *GhM_D03G0127*, *GhM_A05G0343*, *GhM_D12G3197*), CAT (catalase isozyme 1, *GhM_D01G1104*, *GhM_A01G1145*) and CHIT (chitinase/endochitinase, *GhM_A06G0605*, *GhM_D06G0603*, *GhM_D01G2128*, *GhM_A01G2155*) were enriched in the MAPK signal pathway and showed increased expression in VIGS cotton plant (Figure 6I).

To verify the mechanism of the *GhAL19* gene in response to drought and salt stress, we measured the relative expression of *GhNCED1*, *GhRD22*, and *GhCOR47* in plants of WT-H_2_O, WT-treatments, VIGS-H_2_O, and VIGS-treatments (Figure 6J). The results showed that the expressions of *GhNCED1* and *GhCOR47* were significantly increased in WT-NaCl/WT-PEG cotton and VIGS-NaCl/VIGS-PEG cotton compared to the WT-H_2_O, indicating that the expression of *GhNCED1* and *GhCOR47* genes was induced by NaCl and PEG; moreover, VIGS-*GhAL19* could significantly enhance the induced expression of *GhNCED1* and *GhCOR47*. The expression of *GhRD22* was also upregulated by drought and salt stress, however, the expression of *GhRD22* was only further increased in VIGS-PEG plants while decreasing in VIGS-NaCl, indicating that the silencing of the *GhAL19* gene had no direct effect on the expression of *GhRD22* (Figure 6J). The results coincided with transcriptome data. Furthermore, we measured the ABA content of VIGS-NaCl/VIGS-PEG and WT-NaCl/WT-PEG cotton and then found that the ABA contents were increased in VIGS-*GhAL19* cotton under salt or drought stress (Figure 6K). These findings suggest that *GhAL19* may act as a metabolite hub that regulates drought and salt tolerance by mediating multiple pathways, and negatively regulating drought and salt tolerance.

## 3. Discussion

### 3.1. GhAL Genes Are Highly Evolutionarily Conservative in Cotton

The AL gene family contains a conserved alfin domain at the N terminal and a conserved PHD-finger domain at the C terminal. However, unlike most members of the PHD-finger protein family members, the AL genes are only found in plants [15]. They have been reported and studied in higher plants such as Arabidopsis, alfalfa, rice, soybean, corn, western balsam poplar, grape, spinach, and Chinese cabbage, as well as lower plants such as *Chlamydomonas Rhine* [7,9,13,14,15,16,17,18,27]. The analysis of the AL cotton family has not been reported.

As we know, the allotetraploid cotton species *Gossypium hirsutum* cv. NDM8 resulted from the hybridization between two putative diploid cotton species, *G. arboretum* (AA) and *G. raimondii* (DD) [30]. A total of 26 AL genes were identified in NDM8 (Table 1). The number of AL genes in cotton was basically equal to the total sum of *G. raimondii* (13) and *G. arboretum* (13) (Table S2) and greater than that in Arabidopsis (7), rice (9), and poplar (9), suggesting that the AL gene family expands during evolution. Gene duplication plays a key role in the process of gene family expansion, and plants rapidly adapt to new environments after segmental duplication and translocation [31]. Most of the duplicated genes of cotton in our study were driven by purifying selection, as indicated by the Ka/Ks ratio < 1 (Table S1), suggesting the functions of duplicated GhAL genes were highly conserved during subsequent evolutionary events, which could eliminate deleterious loss-of-function mutations. Therefore, a new duplicated gene at both duplicate loci could be fixed and enhanced after purifying selection [32].

It is noteworthy that while the exon/intron arrangements of the majority of homologous gene pairs remained unchanged, there are some variations observed in poplar; *PtAL5* and *PtAL7* contain 6 exons, while only one gene, *PtAL4*, consists of 8 exons [16]. In this study, most AL genes contain four introns and five exons, while *GhAL1*, *GhAL18*, and *GhAL5* contain 4 exons (Figure 2). Analysis of the AL gene structure of *A. thaliana*, *T. halophila*, Chinese cabbage [9,18], and other plants show that almost all AL genes contain four introns and five exons, which is an evolutionarily conserved gene structure.

### 3.2. AL Gene Family Expression Analysis

The AL genes in cotton displayed lower expression levels in different tissues, and the *GhAL6* and *GhAL19* genes showed a high accumulation during ovule development (−3 to 25 DPA), suggesting that the two genes may have significant roles in ovule development and contribute to the plant’s growth and reproduction. In Figure 4B, most AL genes are up-regulated when exposed to drought, salt, or temperature stresses, and their expression would increase over time, especially *GhAL6* and *GhAL19* in Group III. In fact, most of the differences in gene expression are mostly determined by various cis-acting components in the promoter region, which are crucial for their regulatory functions under different environmental stresses [33,34]. Previous studies on rice, poplar, and Chinese cabbage [16,18] found several cis-elements related to abiotic stress response in the AL genes, such as ABRE, AC-I, ARE, as-1, DRE1, ERE, GC-motif, GRE (gibberellin-responsive element), LTRE, MBS, MeJA, MYB, MYC, STRE (Stress Response Element), TCA-element (salicylic acid responsiveness), TC-rich repeats (defense and stress responsiveness), and WRE, and we just found the same cis-elements in our study. Additionally, the promoters of AL genes in Group III contain a greater abundance of cis-acting elements, suggesting that these genes in Group III may play a more significant role in stress resistance mechanisms.

Previous studies have shown AL genes have specific expression patterns, and different ALs have different functions in plant stress resistance [9,14]. It was found that *AtAL3* and *AtAL7* in *A. thaliana* negatively regulated plant salt tolerance, while *AtAL5* increased plant salt tolerance [9,14], and drought stress induced the up-regulation of *AtAL1* gene expression in mature leaves [35]. In *A. hortensis*, *AhAL1* enhances salt and drought stress tolerance in transgenic Arabidopsis plants; the other three AhAL genes cause hypersensitive responses to salt stress in plants [15]. In *P. trichocarpa*, five of nine PtAL genes show slightly higher expression as the stress period progresses, of which, *PtAL4* and *PtAL6* have negative expression during the drought and salt treatment, and the other PtAL genes show non-significant expression compared to the control [16]. Coincidentally, the expression patterns of AL genes in *B. rapa* and maize are found to be similar to those of PtAL genes under stress conditions, suggesting that the role of AL genes in stress response is conserved across different plant species [18,36]. In addition, the response of some OsALs in rice is different at the seedling stage and adult stage under drought treatments. In detail, the *OsAL7.1*, *OsAL7.2*, and *OsAL11* genes show differential expression in response to drought between upland and lowland at the adult stage [27], suggesting that the response and function of OsALs are different at different developmental stages, and the genetic diversity of OsALs further complicates matters [27]. In our study, the expression of six GhAL genes was very low and slight, and the expression of the other 20 GhAL genes was up-regulated under NaCl and PEG treatments at a certain time. Of which, the genes in Group III were more significantly increased under stress, and *GhAL19* and *GhAL6* were outstanders of the genes (Figure 4). Further, VIGS-*GhAL19* in NDM8 enhanced salt and drought treatment tolerance but reduced hot treatment tolerance with the increase in antioxidant enzymes. These results were consistent with the findings of *AtAL7* and *PtAL4/6*. Clearly, the AL gene family must play an important role in growth and stress resistance in cotton, as well as in other plants.

### 3.3. GhAL19 Response to Drought and Salt Resistance

Salt, drought, and temperature stress limit the productivity and geographical distribution of crops worldwide [1]. The AL genes are critical for plant adaptation to environmental stresses like salt, drought, and cold stress [14,15,18]. Recent research shows that drought and salt stresses result in osmotic stresses, leading to the overproduction of ROS, which in turn causes damage to lipids, proteins, and nucleic acids, as well as programmed cell death [36]. However, transgenic plants have been found to improve tolerance to drought, osmotic stress, and salt stress by scavenging ROS [16,37]. The level of MDA is an indicator of ROS effects in stress conditions [8], which is significantly reduced in transgenic plants overexpressing the AL gene during salt and osmotic stresses [7,38]. In this study, the response to drought and salt stresses was determined by measuring MDA levels in VIGS-*GhAL19* lines and WT plants. The results were consistent with previous reports [39,40,41] and suggested that VIGS-*GhAL19* might enhance drought and salinity tolerance by changing MDA levels in silenced plants.

Recently, studies have shown that AL TFs play key roles in plant responses to abiotic stresses with the potential tolerance mechanisms related to ABA signaling [39]. Previous research found that *AhAL1* promoted ABA-induced stomatal closure in response to drought and specifically bound to the promoter regions of genes involved in ABA signaling, such as *PP2C* (protein phosphatase 2C), *DREB1C*, and *GRF7* (growth-regulating factor 7), and then led to the activation of some ABA stress-responsive genes to improve plant adaptation to adverse environments [15]. As we know, the NCED is essential for ABA biosynthesis under abiotic stress, and its expression is induced by abiotic stress [42,43,44]. For example, *OsNCED5* in rice increases ABA levels and enhances tolerance to salt and drought stresses [45]. In our study, we also found the four important genes (NCEDs) (Figure S5) that could activate ABA-dependent and independent stress-responsive genes. There were multiple motifs related to abiotic stress and cis-element GTGGNG (Table S3 and Figure S5) in the promoters of 4 NCEDs. While the cis-element GNGGTG/GTGGNG exactly binds to the conserved domain of AL proteins at the N terminal [7,14]. This suggested that *GhAL19* might directly bind to the promoters of NCEDs and regulate their expressions. Combining the results of transcriptome data and RT-qPCR, it is not difficult to find that *GhAL19* inhibits NCED gene transcription in response to osmotic stress by directly binding to the promotor of NCED.

ERF (ethylene-responsive element binding factor) can regulate drought and salt tolerance through abscisic acid signaling and reactive oxygen species scavenging processes [29,46,47]. In Arabidopsis, overexpression of *AtERF019*, *AtERF1*, and *AtERF74*, respectively, leads to markedly enhanced tolerance to abiotic stresses [48,49,50]. In detail, ERF74 directly binds to the promoter of RbohD and activates its expression under various abiotic stresses. Moreover, the induction of stress marker genes and ROS-scavenging enzyme genes under different stress conditions depends on the ERF74-RbohD-ROS signal pathway [50]. In our research, we found 30 ERFs up-regulated in VIGS-*GhAL19* plants (Figure S6B). Plenty of motifs related to ABA and ethylene (ET) signal pathways were predicted in ERF gene promoters, such as ABRE, ERE, MYB, and MYC motifs (Figure S6C). Of these, 21 ERF genes contain one or more GTGGNG/GNGGTG motifs in their promoter regions (Figure S6D). This means that GhAL19 may also directly regulate ERF genes; thereby, *GhAL19* could regulate plant responses to osmotic stress through ABA- or ER-dependent metabolic pathways.

Therefore, as we know, the *ABI1* and *ABI2* genes encode two PP2C proteins that act as negative regulators of ABA response [51], and the *ABI4* and *ABI5* genes are the best-characterized positive regulators of ABA signaling [52,53]. Several reports have shown that some transgenic plants increase stress tolerance with downregulation of ABI1/2 [54]. Contrary phenotypes of drought tolerance by regulating ABI1/2 have also been reported over the past several years. For example, overexpression of *GmbZIP44*, *GmbZIP62*, or *GmbZIP78* enhanced tolerance to abiotic stress, which may play an important role in ABA signaling by upregulation of *ABI1* and *ABI2* [55]. Overexpression of *GsWRKY20* and *GmWRKY16* in Arabidopsis increased the tolerance to drought stress by regulating *ABI1/2* and downregulating *ABI4/5* [39,56,57]. In our research, *ABI1* and *ABI2* were upregulated, whereas *ABI5* was downregulated in silenced lines under drought and salt stress (Figure 6H and Table S3). Furthermore, a group of LEA genes that play important roles in dehydration tolerance have also been investigated in studies of responses to abiotic stress [58,59], and the expression of LEA14 genes was also upregulated under salt and drought stress in this study (Table S3).

All of these results suggest that VIGS-*GhAL19* in NDM8 improves drought and salt tolerance by regulating the expression levels of ABA signaling and stress-related genes. Similar observations were found in other plant species [60,61,62,63].

## 4. Conclusions

**Figure 7:**
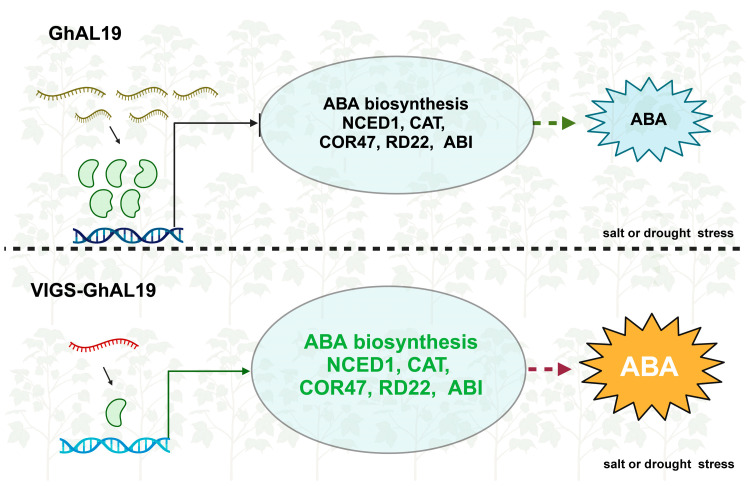
A proposed model of transcription factor *GhAL19* regulates drought and salt tolerance in cotton. *GhAL19* is expressed slightly under normal conditions, whereas induced by drought and salt tolerance. VIGS-*GhAL19* gene could upregulate the content of ABA via regulating the expression levels of ABA biosynthesis genes (like *NCED1*, *COR47*, *RD22*, et al.), thereby negatively regulating drought and salt stresses.

This is the first study to discuss the relationship between the Alfin-like gene and osmotic stress in cotton. The function of *GhAL19* is demonstrated through RNA-seq, VIGS experiments, and physiological index determination, highlighting the *GhAL19* novel role in osmotic stress. *GhAL19* has been proven to negatively regulate the tolerance of drought and salt by affecting related genes involved in the ABA signal pathway (Figure 7).

These findings provide mechanisms for the early plant response during drought and salt stress before ABA accumulation. However, the molecular network of *GhAL19* is still unclear at present, and more experiments are needed to elucidate the detailed molecular mechanisms, such as the validation of downstream target genes of the *GhAL19* gene and its response to exogenous ABA.

## 5. Material and Methods

### 5.1. Plant Materials and Treatment

*G. hirsutum* cv. NDM8 plant materials were used in this research. The seeds of NDM8 were soaked in distilled water for 1 day and then germinated on wet gauzes for another day at 25◦ C. Germinant seeds were transferred to water and inserted in a foam panel under the following conditions: 25 °C, 16 h/8 h (day/night), 6000–6500 Lux light, and 60–70% humidity for NaCl and PEG treatments; the hot treatments were conducted at a constant temperature of 37 °C through all day with 16 h/8 h (day/night), 6000–6500 Lux light and 80% humidity. The seedlings were divided into two groups for each treatment when the two cotyledons fully unfolded and the true leaves did not grow. One group was watered with 1/2 MS nutrient solution and water, while the other group was watered with 1/2 MS nutrient solution containing 160 mM NaCl [64] for 3 days or a 15% PEG6000 [65] solution for 6 days; in addition, 1/2 MS nutrient was used as a solution for all the hot treatments for 8 days.

### 5.2. Identification and Property Analysis of AL Genes

The AL genome datasets of cv. NDM8 and *A. thalianas* were downloaded from Cottongen (https://www.cottongen.org (accessed on 24 May 2024)) and TAIR 10 (http://www.arabidopsis.org/ (accessed on 24 May 2024)), respectively. The AL genome datasets of other plant species were downloaded from NCBI (https://www.ncbi.nlm.nih.gov/ (accessed on 24 May 2024)). The SMART (http://smart.embl.de/) database was used to confirm the conservation of AL proteins (PF12165). The molecular weight (MW), and isoelectric point (pI) of each GhAL were obtained from CottonFGD (https://cottonfgd.net/ (accessed on 24 May 2024)). Cell-PLoc 2.0 (http://www.csbio.sjtu.edu.cn/bioinf/euk-multi-2/ (accessed on 24 May 2024)) was for subcellular localization prediction [66]. All AL gene names and their IDs are listed in Table 1.

### 5.3. Multiple Alignments and Phylogenetic Analysis

The amino acid sequences of the AL gene family were compared multiple times using MEGA 7.0 software [67], and the sequences were then visualized using DNAMAN version 8.0.8.789. To construct the phylogenetic tree, the MEGA 7.0 software and the Neighbor-Joining (NJ) method were utilized. Additionally, 1000 bootstrap replicates were performed on each node to ensure reliability [68].

### 5.4. Gene Structure, Motif Distribution, and Promoter Cis-Elements Analysis

MEME (https://meme-suite.org/meme/tools/meme (accessed on 24 May 2024)) database was employed to search conserved motifs, with a limit of 12 motifs and other default parameters [69]. Next, 2000 bp promoter sequences of GhAL were obtained from CottonFGD for analysis of cis-elements [70].

PlantCARE (http://bioinformatics.psb.ugent.be/webtools/plantcare/html/ (accessed on 24 May 2024)) database was used to predict gene promoter motifs [71]. The exon/intron information and gene location information were fetched from GFF3 files and subsequently visualized using TBtools-IIv2.096 [72].

The NCBI CDD (https://www.ncbi.nlm.nih.gov/Structure/bwrpsb/bwrpsb.cgi (accessed on 24 May 2024)) was used to identify the Alfin-like protein domain [73]. Then all the data above were mapped by using Tbtools-IIIIv2.096 [72].

### 5.5. Chromosomal Location, Gene Duplication, and the Calculation of Ka, Ks, and Ka/Ks Values

Segmental and tandem duplications were detected by MCScanX with default parameters [74]. The duplication events were fetched and then displayed with TBtools [72]. Homologous genes between At- and Dt-subgenomes were determined using the bidirectional best-hit method in BLAST. We used MAGA7.0 [68] to construct protein-coding DNA alignments. Then the paired sequences were used to calculate Ka, Ks, and Ka/Ks values using TBtools-IIIIv2.096 [72].

### 5.6. AL Genes Expression Patterns under Abiotic Stress

To analyze the expression pattern of the AL genes under abiotic stress, we obtained high-throughput RNA-seq data of leaf tissue under control and four stress treatments (NaCl stress, PEG stress, 37 °C stress, and 4 °C stress) from CottonMD (https://yanglab.hzau.edu.cn/CottonMD/download.1 (accessed on 24 May 2024)). Subsequently, cluster heat maps were drawn to visualize the expression pattern by TBtools-IIv2.096 [72].

### 5.7. Virus-Induced Gene Silencing Assay

The Cotton VIGS technique was implemented following the protocol published by Gao [75]. A specific fragment of target genes, approximately 276 bp in length, was synthesized and inserted into the vector pTRV2 using *Bam*H I and *Kpn* I sites. The cloroplastosalterados 1 (CLA1) gene was used as a reliable marker to evaluate gene silencing. The experiments were carried out using at least 30 plants per treatment and repeated three times.

### 5.8. RNA Isolation and RT-qPCR

The Total RNA of seeding roots was extracted using the TIANGEN-RNAprep Pure Plant Plus Kit (Tiangen, Beijing, China), and Quantitative real-time PCR (RT-qPCR) was performed using the TB Green^®^ Fast PCR Mix (TaKaRa, Dalian, Japan). The UBQ gene (XM 016855771.2) of cotton was used as an internal reference gene. Gene expression levels were calculated by using the 2^−ΔΔCT^ method [76]. Three biological replicates and three technical replicates were set for each treatment. The primers used for RT-qPCR are listed in Table S4.

### 5.9. Determination of SOD, POD Enzymes Activities and Malondialdehyde, ABA Content

The cotton root samples collected from the WT treatment (WT-NaCl/WT-PEG/WT-37 °C) and the VIGS-*GhAL19* lines (VIGS-NaCl/VIGS-PEG/VIGS-37 °C) to measure the levels of malondialdehyde and antioxidant enzyme activities. Peroxidase assay kit (POD), superoxide Dismutase (SOD) assay kit, and Malondialdehyde (MDA) assay kit were provided by Nanjing Jiancheng Bioengineering Institute.

To determine the concentration of the plant hormone abscisic acid (ABA), we followed the method outlined in the ABA ELISA kit. This involved drawing a standard curve and calculating the results based on it.

### 5.10. RNA-Seq Analysis

Total RNA was extracted from the root tissues of WT treatment (WT-NaCl/WT-PEG) and VIGS-*GhAL19* lines (VIGS-NaCl/VIGS-PEG) during the phenotypic exposure after the plant was exposed to drought and salt stress. Sequencing was performed on the Illumina HiSeq2500 plat by the Majorbio (Shanghai, China) company. The reads filtered above were then mapped to the cotton cv. NDM8 (https://cottonfgd.net/ (accessed on 24 May 2024)) reference genome using TopHat2 v2.1.1 software [77]. The expression values of genes were presented as Transcripts Per Kilobase of exon model per Million mapped reads (TPM) values using RSEM. Eventually, log_2_(1 + TPM) was calculated as the average of two replicates. Subsequently, we performed KEGG annotations, and KEGG enrichment analysis was performed using Majorbio Cloud (https://cloud.majorbio.com/ (accessed on 24 May 2024)).

### 5.11. Statistical Analysis

All experiments were repeated three times. Statistical analysis was performed using Graph Pad Prism^®^8.3.0 (Graph Pad, San Diego, CA, USA). Analysis of variance (ANOVA) was used to determine the statistical significance of the difference between the treatment and control groups (*p*-value < 0.05).

## Figures and Tables

**Table 1:** Characteristics of Alfin-like gene and predicted properties of Alfin-like proteins in NDM8.

Gene Name	Gene ID	Genomics Position	Genomic DNA Length (bp)	CDS	AA Siza	Mw (kDa)	pI	Predicted Subcellular Location
*GhAL1*	*GhM_A03G1160.1*	A03: 37,971,435–37,979,034	7600	876	291	32.82	4.992	Nucleus. Extracell.
*GhAL2*	*GhM_A04G1187.1*	A04: 77,167,270–77,170,430	3161	714	237	26.76	5.419	Nucleus
*GhAL3*	*GhM_A05G0428.1*	A05: 4,040,221–4,043,183	2963	759	252	28.48	4.84	Nucleus
*GhAL4*	*GhM_A05G1804.1*	A05: 15,784,576–15,789,887	5312	759	252	28.35	4.894	Nucleus
*GhAL5*	*GhM_A06G0891.1*	A06: 19,353,566–19,362,999	9434	531	176	20.02	4.347	Cytoplasm, Extracell.
*GhAL6*	*GhM_A06G1028.1*	A06: 28,076,813–28,080,126	3314	762	253	28.58	5.131	Nucleus
*GhAL7*	*GhM_A06G1365.1*	A06: 58,493,853–58,499,312	5460	759	252	28.39	4.889	Nucleus
*GhAL8*	*GhM_A06G2137.1*	A06: 119,744,982–119,747,574	2593	714	237	26.99	6.127	Nucleus
*GhAL9*	*GhM_A07G0239.1*	A07: 2,374,160–2,376,293	2134	735	244	27.2	4.541	Nucleus
*GhAL10*	*GhM_A10G0582.1*	A10: 5,737,930–5,741,746	3817	762	253	28.58	5.13	Nucleus
*GhAL11*	*GhM_A10G2975.1*	A10: 117,448,657–117,451,590	2934	678	225	25.48	4.906	Nucleus
*GhAL12*	*GhM_A13G0186.1*	A13: 1,916,867–1,921,603	4737	714	237	27	5.243	Nucleus
*GhAL13*	*GhM_A13G2539.1*	A13: 106,691,862–106,695,119	3258	765	254	28.49	5.136	Nucleus
*GhAL14*	*GhM_D02G1195.1*	D02: 28,282,641–28,290,842	8202	756	251	28.33	4.898	Nucleus
*GhAL15*	*GhM_D04G1662.1*	D04: 48,884,628–48,887,214	2587	714	237	26.61	5.965	Nucleus
*GhAL16*	*GhM_D05G0448.1*	D05: 3,404,207–3,407,198	2992	759	252	28.41	4.84	Nucleus
*GhAL17*	*GhM_D05G1819.1*	D05: 14,302,797–14,308,076	5280	759	252	28.35	4.894	Nucleus
*GhAL18*	*GhM_D06G0897.1*	D06: 14,139,433–14,143,792	4360	801	266	30.35	6.247	Nucleus
*GhAL19*	*GhM_D06G1025.1*	D06: 18,048,829–18,052,417	3589	762	253	28.68	5.139	Nucleus
*GhAL20*	*GhM_D06G1530.1*	D06: 39,915,003–39,919,254	4252	759	252	28.41	4.889	Nucleus
*GhAL21*	*GhM_D06G2116.1*	D06: 59,847,535–59,850,182	2648	714	237	27.08	5.22	Nucleus
*GhAL22*	*GhM_D07G0240.1*	D07: 2,709,589–2,711,664	2076	711	236	26.79	4.605	Nucleus
*GhAL23*	*GhM_D10G0556.1*	D10: 5,395,887–5,400,299	4413	762	253	28.61	5.13	Nucleus
*GhAL24*	*GhM_D10G2926.1*	D10: 67,932,794–67,936,131	3338	555	184	20.83	6.474	Nucleus
*GhAL25*	*GhM_D13G0175.1*	D13: 1,843,425–1,847,948	4524	714	237	27.06	5.243	Nucleus
*GhAL26*	*GhM_D13G2452.1*	D13: 60,675,626–60,678,409	2784	759	252	28.26	5.136	Nucleus

## Data Availability

The RNA-seq data generated from the silenced cotton and its control plants have been deposited in the NCBI database (PRJNA1117094). All other relevant data can be found within the manuscript and its Supplementary Materials.

## Supplementary Materials

The following supporting information can be downloaded at: https://www.mdpi.com/article/10.3390/plants13131831/s1, Figure S1 The phylogenetic tree was reconstructed with Maximum likelihood (ML) method. Figure S2 Alignment of conserved Alfin domain and the PHD domain in A. thaliana and NDM8. The genes were all the AL genes in A. thaliana and the AL genes in NDM8 except GhM_A06G0891.1 and GhM_D10G2926.1 because of the two genes had one alfin domain and no PHD domain in NDM8. High consensus color was red, low consensus color was blue, and the neutral color was black. Figure S3 Chromosomal mapping of 26 GhAL genes. Figure S4 Predicted cis-regulatory element in the 2000 bp region upstream of the start codon (ATG) of each GhAL gene. These genes were divided into four groups (Group I/II/III/IV). These elements were divided into two types including stress-responsive elements (AS-1, AC-1, ARE, DRE1, LTR, MBS, MYB, MYC, TC-rich repeats, GC-motif, WRE3, W-box, STRE and WUN-motif), hormone-responsive elements (ABRE, ERE, GARE, P-box, CGTCA-motif, TGACG-motif and TGA-element). Figure S5 The four upregulated NCED genes in VIGS-GhAL19. (A) The ID of upregulated genes of NCED. (B) The heatmap of NCED expressions under salt and PEG stress. Heatmap was normalized by the average log_2_(TPM+1). (C) The number of cis-element and GNGGTG/GTGGNG motif of NCED promoters. Figure S6 The 30 upregulated ERF genes in VIGS-GhAL19. (A) The phylogenetic tree of the GhERF genes. (B) The heatmap of ERFs expressions under salt and PEG stress. Heatmap was normalized by the average log_2_(TPM+1). (C) Predicted cis-regulatory elements and number in the 2000 bp region upstream of the start codon (ATG) of each GhERF gene. (D) The species and quantity of GNGGTG/GTGGNG motifs in each ERF gene promoter. Table S1 Ka/Ks analysis for the duplicated AL gene pairs. Table S2 The details of AL gene family in *Gossypium raimondii* and *Gossypium arboretum.* Table S3 The expression of log_2_ (TPM+1) of genes from transcriptome analysis. Table S4 The primers used in this study.
