# Supplementary figures and images for: Genome-Wide Identification of the Alfin-like Gene Family in Cotton (Gossypium hirsutum) and the GhAL19 Gene Negatively Regulated Drought and Salt Tolerance
Source: Plants (Basel). 2024 Jul 3;13(13):1831. doi: 10.3390/plants13131831 (PMC11243875; doi:10.3390/plants13131831)

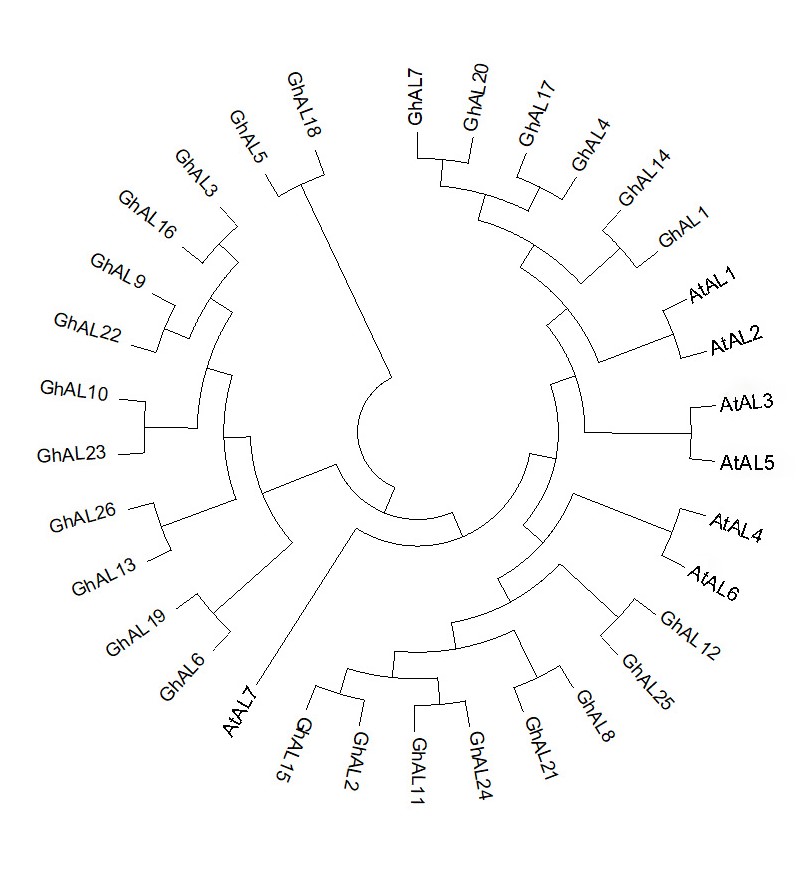

Supplement: Supplementary file 1 [file plants-13-01831-s001.zip › supplementary files/Fig S1.jpg]

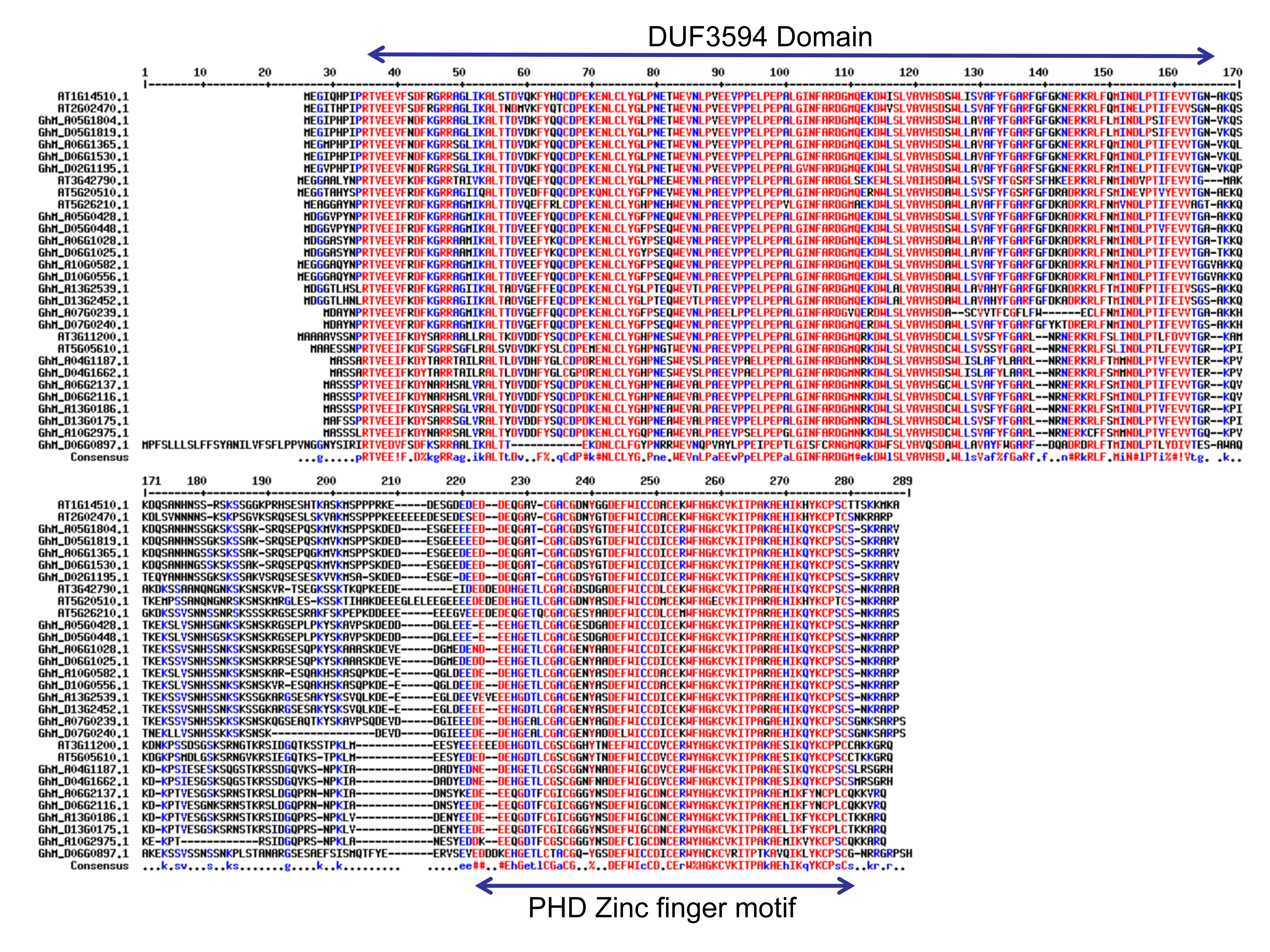

Supplement: Supplementary file 1 [file plants-13-01831-s001.zip › supplementary files/Fig S2.png]

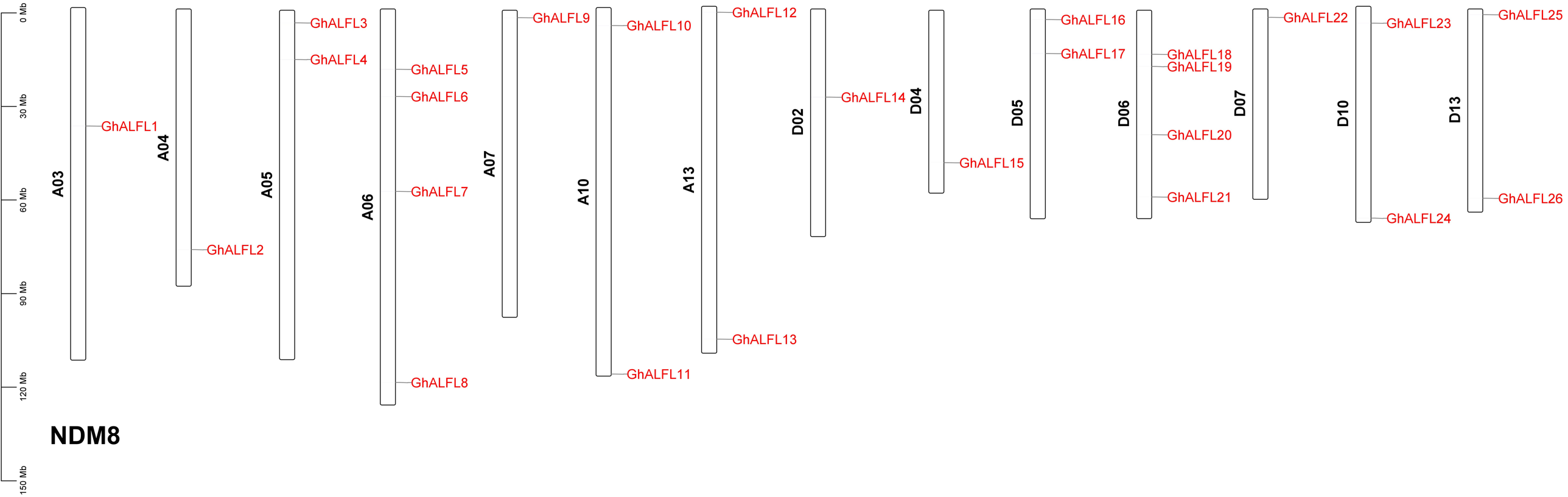

Supplement: Supplementary file 1 [file plants-13-01831-s001.zip › supplementary files/Fig S3.png]

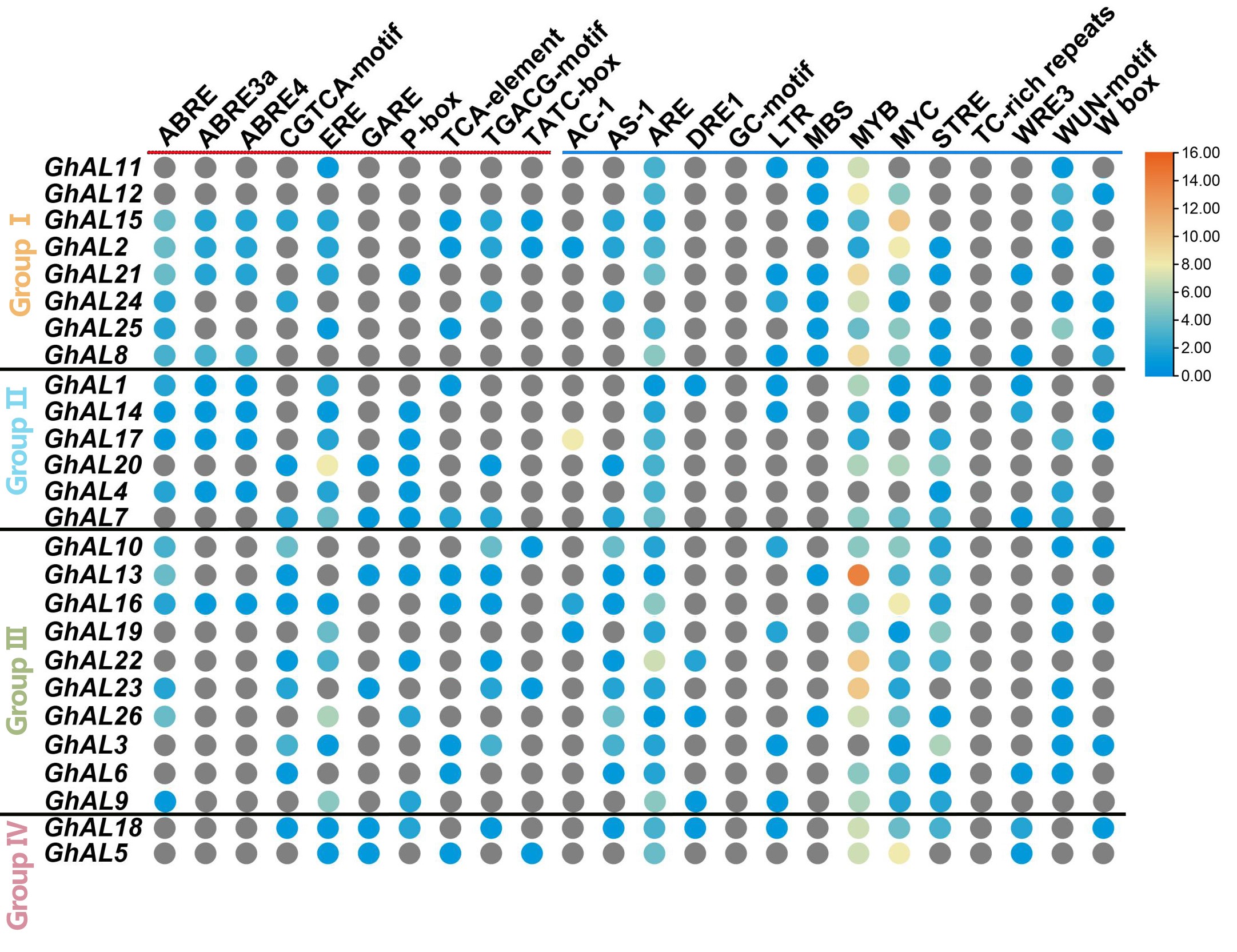

Supplement: Supplementary file 1 [file plants-13-01831-s001.zip › supplementary files/Fig S4.jpg]

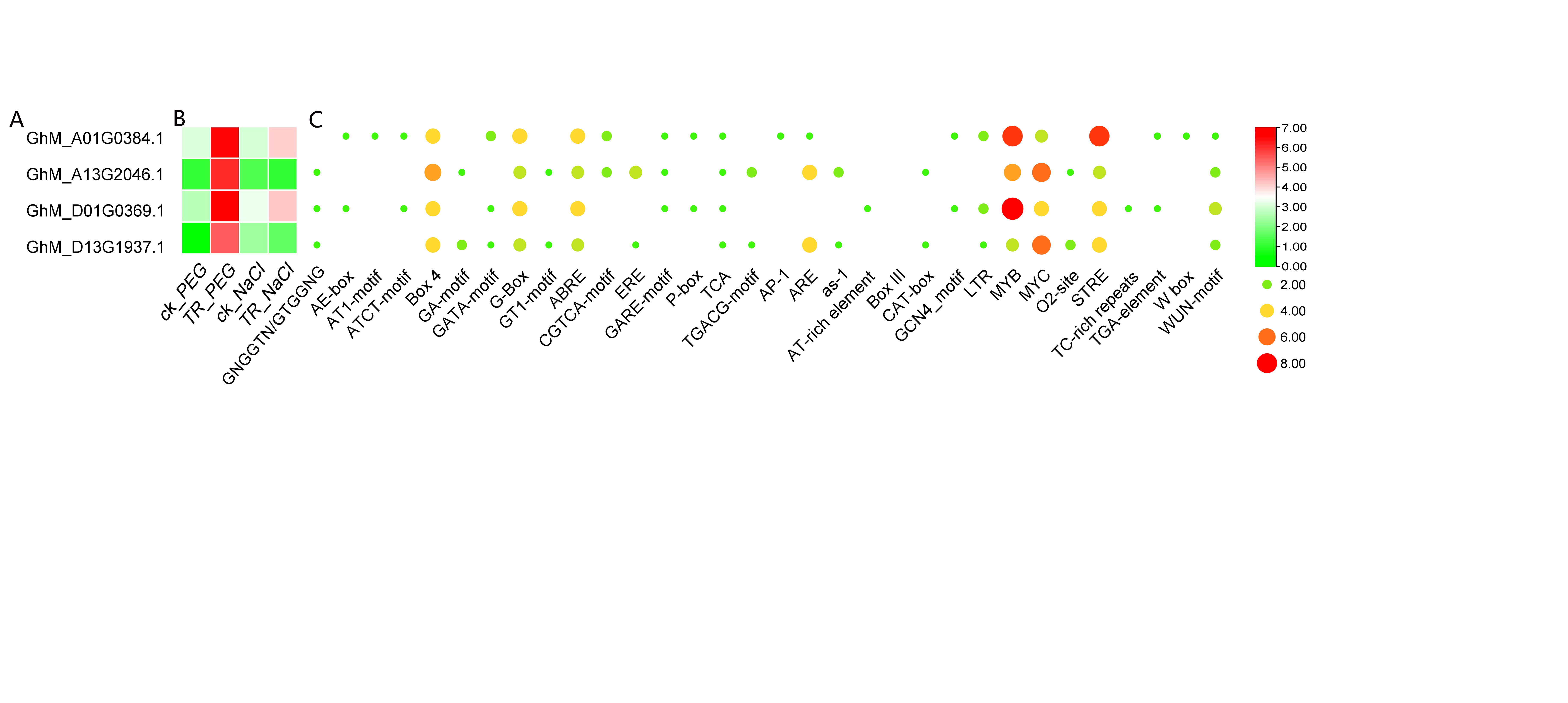

Supplement: Supplementary file 1 [file plants-13-01831-s001.zip › supplementary files/FIG S5 NCED.jpg]

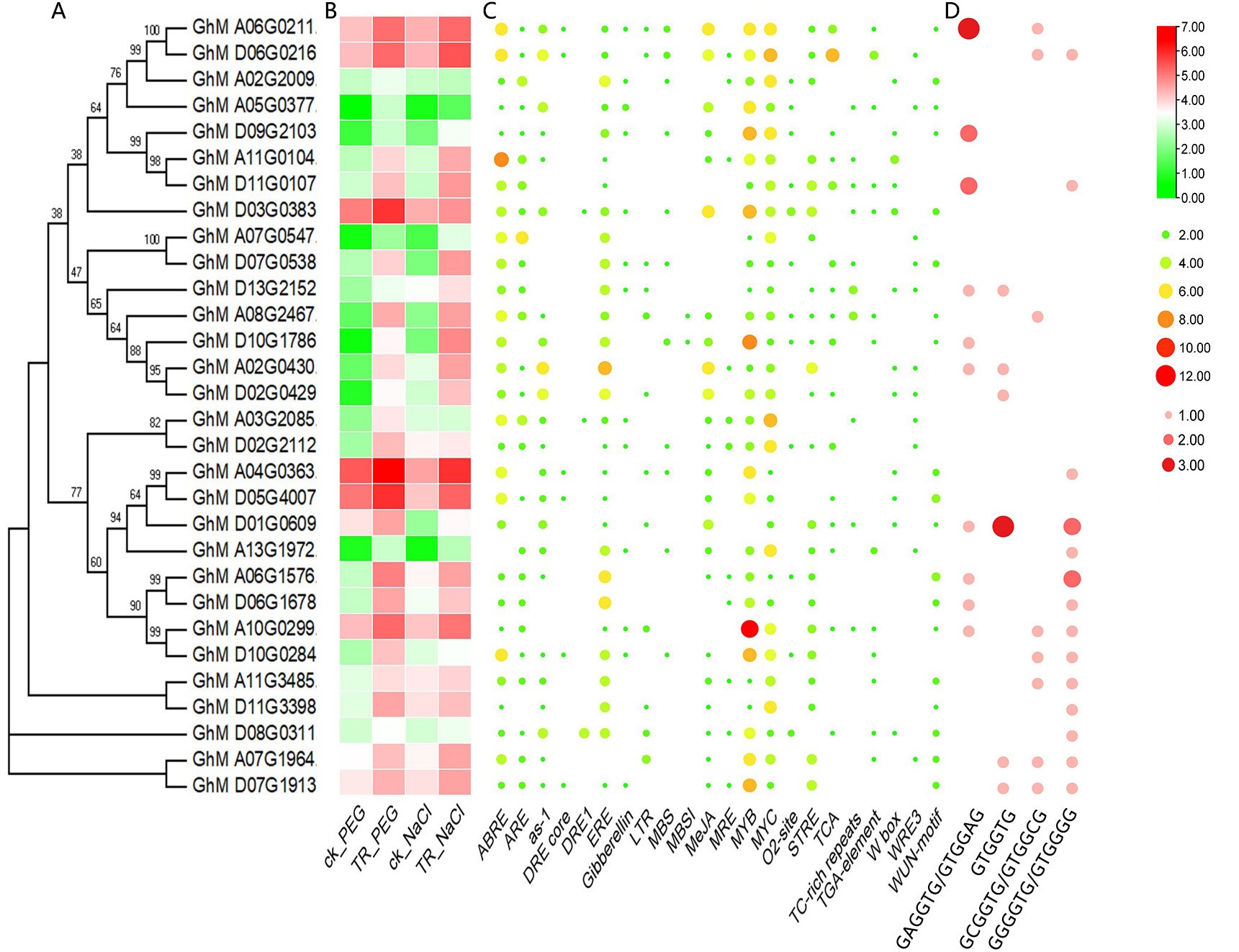

Supplement: Supplementary file 1 [file plants-13-01831-s001.zip › supplementary files/FIG S6 ERF.jpg]
